# Visual Outcomes of Cataract Surgery at a Tertiary Care Hospital in Northern Sri Lanka

**DOI:** 10.7759/cureus.105962

**Published:** 2026-03-27

**Authors:** Malaravan Muthusamy, Kumaran Subaschandren, Kumanan Thirunavukarasu, Aruljenani Kumutharanjan, Powsiga Uruthirakumar, Thurga Jeyaratnam

**Affiliations:** 1 Ophthalmology, Teaching Hospital Jaffna, Jaffna, LKA; 2 Community and Family Medicine, Faculty of Medicine, University of Jaffna, Jaffna, LKA; 3 Medicine, Faculty of Medicine, University of Jaffna, Jaffna, LKA; 4 Center for Digital Epidemiology, Faculty of Medicine, University of Jaffna, Jaffna, LKA

**Keywords:** best corrected visual acuity (bcva), cataract, low and middle-income countries, northern sri lanka, phacoemulsification cataract surgery, post operative outcomes, postoperative visual outcome, world health organization (who)

## Abstract

Background: Cataract is the leading cause of blindness and visual impairment. Cataract surgery is the most common procedure for restoring vision in individuals with cataracts. It is important to assess the surgical outcomes of cataract surgery to ensure the highest standard of vision restoration, improved patient quality of life, and service delivery. Therefore, the present study aimed to evaluate the visual outcomes of cataract surgery among patients attending the Eye Unit of Teaching Hospital Jaffna, Northern Sri Lanka, at the fourth week follow-up postoperatively.

Methods: An institution-based, retrospective analytical study design was conducted among 1,133 patients who underwent cataract surgery using phacoemulsification with intraocular lens implantation. The study participants were selected using a systematic random sampling technique. Data were obtained through a structured data extraction form derived from patients’ medical records and an interviewer-administered questionnaire, and were subsequently analysed using IBM SPSS Statistics for Windows, Version 25 (Released 2017; IBM Corp., Armonk, New York, United States). Proportions, summary statistics, and tables were used to present the findings. Multivariable linear regression was performed to identify associations among the variables. A p‑value of < 0.05 was considered indicative of statistical significance.

Results: Among 1,133 patients who underwent phacoemulsification with intraocular lens implantation, 1,027 (90.6%) achieved good postoperative visual outcomes of 6/12 or better. The majority, 699 (61.7%), were female, and 800 (70.6%) were aged 60 years and above. The most common systemic comorbidities were hypertension with 386 (34.1%) patients and diabetes mellitus with 350 (30.9%), while 24 (2.1%) of them had pre-existing ocular comorbidities. Patients aged 60 years and above demonstrated significantly less improvement in visual acuity than those aged <60 years (p < 0.001). Diabetes mellitus was independently associated with reduced postoperative visual improvement (p = 0.014), whereas sex and pre-existing ocular comorbidities were not.

Conclusion: The visual outcome following cataract surgery exceeds WHO recommendations. In this study, age and diabetes mellitus were associated with postoperative visual outcomes, whereas the presence of other ocular comorbidities and sex were not. Furthermore, this study demonstrates that good postoperative visual outcomes can be achieved with cataract surgery even in resource-constrained settings.

## Introduction

Cataract is characterised by the lens's loss of transparency, which alters its refractive characteristics and increases light scattering, leading to blindness or distorted vision [1]. Cataracts are among the major causes of blindness worldwide, accounting for around 15.2 million cases, or 45% of total blindness [2]. Furthermore, it is expected to rise to 120 million by 2030, driven by population growth and an ageing global population [3]. According to Sri Lanka's population-based survey, the only nationwide survey to date, cataracts account for 79% of all cases of visual impairment [4]. The impact of cataracts on vision loss, particularly in the geriatric population, can be substantial, negatively affecting an individual's quality of life by impairing physical and mental health and interfering with everyday activities and independent living [5]. Consequently, it adds burden to the inexorable cycle of poverty and causes significant social and economic consequences, particularly in underprivileged regions.

Cataract surgery remains the only effective surgical procedure globally for restoring visual function [6]. The cataract surgical rate (CSR), which measures the number of procedures performed per million people in a given year, is the most relevant public health metric for determining access to cataract services. Despite being proven to be one of the most cost-effective health interventions, cataract surgery may result in poor visual outcomes. This could be due to surgical complications, ocular comorbidities, or inadequate optical correction, even with intraocular lens (IOL) implantation [7]. The primary method for evaluating cataract surgery outcomes has been to measure changes in postoperative visual acuity. This simple, direct metric can provide a good indication of the quality of cataract surgery and postsurgical treatment. According to the latest updates (2024-2026) released by the World Health Organisation (WHO), the outcomes of cataract surgery can be measured in three categories: "Good" (postoperative visual acuity 6/12 or better); "Borderline" (visual acuity between 6/12 and 6/60); and "Poor" (visual acuity <6/60) [8]. The WHO recommendations for acceptable outcomes, assessed between four and 12 weeks postoperatively, state that at least 85% of cases should have a good outcome and that less than 5% should have a poor outcome. Further, the Global Initiative for the Elimination of Avoidable Blindness emphasises that cataract surgeries should achieve high success rates in terms of visual acuity and improved quality of life [9].

Even though Sri Lanka is a low- to middle-income country, phacoemulsification is the most commonly performed cataract surgical technique in both government and private hospitals. Cataract surgery outcomes in Sri Lanka have improved significantly, driven by advancements in surgical technology and community initiatives. However, there is a dearth of national data on cataract surgery coverage and outcomes, with data available only from one province [10].

Northern Sri Lanka, one of the provinces with a predominantly rural population, is a post-conflict, resource-constrained region. It is reported to have the highest prevalence of blindness and visual impairment, with cataract as the major cause [11]. Nevertheless, the region has already achieved a CSR of 10/1000 in 2023 and 15/1000 in 2024, according to unpublished hospital data [12]. Even so, information about surgery outcomes remains elusive, hampering cataract prevention and control in resource-constrained settings. Furthermore, as far as we are aware, this is the first study to evaluate visual acuity quality after cataract surgery in the Northern Province. Therefore, the present study aimed to evaluate the visual outcomes following cataract surgery among patients attending the Teaching Hospital Jaffna, focusing on the primary outcome of postoperative best-corrected visual acuity (BCVA) at four weeks post-surgery, classified according to WHO criteria. In addition, the study evaluated factors associated with postoperative visual improvement.

## Materials and methods

Study design and setting

This was a hospital-based, retrospective analytical study of patients who had undergone cataract surgery at the Eye Unit of Teaching Hospital Jaffna, conducted from February 2025 to November 2025. Teaching Hospital Jaffna is the only tertiary hospital in Northern Sri Lanka, and the Eye Unit of Teaching Hospital Jaffna serves as the major referral centre providing both routine and high-volume cataract surgical services to the broad catchment population [12]. This Eye Unit operates with less than 50% of the WHO’s recommended human and equipment resources. It is equipped with its own surgical unit, capable of performing 200-230 cataract surgeries per day by utilising the available resources effectively. All patients underwent phacoemulsification surgery with the implantation of monofocal, hydrophobic, foldable IOLs. Multiple skilled surgeons with varying levels of experience performed surgeries following a standardised surgical protocol.

Eligibility criteria

Patients aged 18 years and above who underwent cataract surgery in the Eye Unit of Teaching Hospital Jaffna during the study period and attended the routine postoperative follow-up visit at the fourth week, with complete preoperative and postoperative data, were included in the study. Patients with pre-existing ocular pathologies other than cataract that could affect postoperative visual outcomes, incomplete or missing preoperative/postoperative data, and those with combined surgeries, were excluded. Patients were chosen from the cataract surgery registry, and their medical records were retrospectively examined.

Sample size and sampling technique

The sample size for this study was determined using the single population proportion formula, based on an expected proportion of poor visual outcomes after cataract surgery of 12.1%, as reported in a previous study [13], with a 95% confidence level and a 2% margin of error. To account for potential non-response, the calculated sample size was adjusted, resulting in a total of 1,133 participants. Participants were recruited using a systematic random sampling method, where the sampling interval was calculated by dividing the total number (n = 5668) of eligible patients who visited the eye unit during the study period by the desired sample size, resulting in a sampling interval of five. Accordingly, the first participant was randomly chosen using a lottery method, and every fifth patient afterwards was included in the study. Ethical clearance sought from the Ethical Review Committee of the Faculty of Medicine, University of Jaffna (J/ERC/25/171/NDR/0343).

Data collection

Data were extracted from medical records and the surgical registry using a structured data extraction form derived from the WHO cataract surgery outcome monitoring form [14], along with an interviewer-administered brief questionnaire for socio-demographic information that is not routinely recorded in medical records. The interviewer-administered questionnaire consisted of variables such as age, sex, ethnicity, religion, and employment status, and was obtained at the time of patient registration. The information was documented in the clinical records. Clinical variables such as preoperative and postoperative visual acuity, systemic comorbidities, ocular comorbidities, and surgical details were extracted from these records using a standardised data extraction form.

Outcome measures

Primary outcome variable of the study was BCVA at the fourth week follow up, whereas the secondary outcome variables were postoperative complications, explanatory variables including sociodemographic characteristics such as age, sex, ethnicity, religion, and occupation; pre-operative clinical characteristics such as BCVA, and presence of other ocular comorbidities (ocular comorbidities, which were not identified during the routine preoperative comprehensive assessment and were documented in the medical records during follow up including certain macular and optic nerve pathologies), and systemic comorbidities such as diabetes mellitus, hypertension, dyslipidemia, and ischemic heart disease and side of the operated eye. Adverse events affecting the operated eye within the first four weeks after surgery were classified as postoperative complications, including corneal issues, infections, or other clinically significant events documented in the patient’s medical record. Intraoperative complications were not part of this study's analysis. Informed consent was obtained from all eligible patients included in the study before data collection.

Visual acuity measurement

Visual acuity was measured using Snellen charts, and the results were converted to the logarithm of the minimum angle of resolution (LogMAR) scale for analytical purposes. Thus, 6/6 vision corresponded to a score of 0.0 logMAR, 6/12 to 0.3 logMAR, and so on. Counting fingers vision was recorded as 2.1 logMAR, hand movement vision was recorded as 2.4 logMAR, and light perception was recorded as 2.7 logMAR. BCVA was selected as the primary outcome. Further, visual acuity measurements were classified according to the International Classification of Diseases 11th Revision (2025) classification for distance vision impairment as follows: mild vision impairment, where visual acuity is worse than 6/12, 20/40, or 0.5, but equal to or better than 6/18, 20/70, or 0.3; moderate vision impairment where visual acuity is worse than 6/18, 20/70, or 0.3, but equal to or better than 6/60, 20/200, or 0.1; severe vision impairment where visual acuity is worse than 6/60, 20/200, or 0.1, but equal to or better than 3/60, 20/400, or 0.05; blindness where visual acuity is worse than 3/60, 20/400, or 0.05 [15].

The outcomes of the cataract surgeries were defined considering the visual acuity of the best eye, according to the WHO's latest guidelines for monitoring surgery outcomes, which categorise the outcomes as “good” when visual acuity is 0.3 or better, “borderline” when visual acuity is between 0.3 and 1.0, and “poor” when acuity is less than 1.0 [8].

Statistical analysis

Descriptive statistics were used to summarise patient characteristics and visual outcomes. Categorical variables were presented as frequencies and percentages, while continuous variables were summarised using means and standard deviations. Multiple linear regression was performed to identify factors associated with changes in visual acuity following cataract surgery because the main dependent variable was continuous. The associated factors included for linear regression analysis were age, sex, preoperative visual acuity, presence of ocular comorbidities, and comorbidities. A p-value <0.05 was considered statistically significant. IBM SPSS Statistics for Windows, Version 25 (Released 2017; IBM Corp., Armonk, New York, United States) was used for the statistical analysis.

## Results

This retrospective analytical study included 1,133 eyes (1,133 patients) who underwent cataract surgery. Phacoemulsification with IOL implantation was performed by multiple skilled surgeons in all 1,133 eyes. The mean age was 64.5 ± 8.97 years, ranging from 28 to 93 years, despite the inclusion criteria encompassing all adults aged 18 years and above. Of these patients, females accounted for the majority (699, 61.7%), whereas males accounted for 634 (38.3%). Further, 333 (29.4%) were under 60 years old, while 800 (70.6%) were above 60 years old. The majority of patients were Sri Lankan Tamils with 1,123 (99.1%). A total of 973 patients (85.9%) identified as Hindu, followed by 145 (12.8%) Christians, 10 (0.9%) Muslims, and five (0.4%) Buddhists. Furthermore, 281 (24.8%) were self-employed, 61 (5.4%) were employed full-time, four (0.4%) were unemployed, 174 (15.4%) were retired, and 613 (54.1%) were homemakers (Table 1).

**Table 1:** Demographics and Clinical Characteristics of Patients (n = 1,133)

Characteristics	Frequency (n)	Percentage (%)
Age group		
Below 60 years	333	29.4
60 years and above	800	70.6
Sex		
Male	634	38.3
Female	699	61.7
Ethnicity		
Sri Lankan Tamils	1,123	99.1
Sinhalese	2	0.2
Sri Lankan Moors	8	0.7
Religion		
Hindu	973	85.9
Christians	145	12.8
Muslim	10	0.9
Buddhist	5	0.4
Job status		
Self-employed	281	24.8
Full-time employee	61	5.4
Unemployed	4	0.4
Retired	174	15.4
Homemaker	613	54.1
Eye operated		
Right	613	54.1
Left	520	45.9
Systemic comorbidities		
Diabetes mellitus	350	30.9
Hypertension	386	34.1
Dyslipidemia	331	29.2
Ischemic heart disease	98	8.6
Ocular comorbidities		
Presence of other ocular comorbidities	24	2.1

Furthermore, 613 (54.1%) underwent surgery on the right eye and 520 (45.9%) on the left eye. The most common systemic illness among the patients in this study was hypertension, which was present in 386 (34.1%) individuals, followed by diabetes mellitus at 350 (30.9%), dyslipidaemia at 331 (29.2%), and ischemic heart disease at 98 (8.6%). In addition, 24 (2.1%) of them had presented with ocular comorbidities other than cataracts.

Preoperatively, 52 (4.6%) patients had a visual acuity of ≥6/12, 157 (13.9%) between <6/12 and ≥6/18, 461 (40.7%) between < 6/18 and ≥ 6/60, 108 (9.5%) between < 6/60 and ≥ 3/60, and 355 (31.3%) with <3/60. At the fourth-week postoperative follow-up, 1,027 (90.6%) patients had a visual acuity of ≥6/12, 37 (3.3%) had visual acuity between <6/12 and ≥6/18, 51 (4.5%) between <6/18 and ≥6/60, three (0.3%) between <6/60 and ≥3/60, and 15 (1.4%) had <3/60 (Table 2).

**Table 2:** Comparison of Preoperative and Fourth-Week Postoperative BCVA (n = 1,133) LogMAR values corresponding to count fingers (CF), hand movements (HM), and perception of light (PL) were substituted with 2.10, 2.40, 2.70, and 3.00 logMAR, respectively [16]. BCVA: best-corrected visual acuity

Visual Acuity			Preoperative		Postoperative at the Fourth Week	
Snellen (Metric - 6m)	Snellen (Imperial - 20ft)	LogMAR	Frequency	Percentage (%)	Frequency	Percentage (%)
6/6	20/20	0	-	-	153	13.5
6/9	20/30	0.18	14	1.2	730	64.4
6/12	20/40	0.30	38	3.4	144	12.7
6/18	20/60	0.48	157	13.9	37	3.3
6/24	20/80	0.60	178	15.7	27	2.4
6/36	20/120	0.78	119	10.5	9	0.8
6/60	20/200	1.0	164	14.5	15	1.3
5/60	20/240	1.08	14	1.2	1	0.1
4/60	20/300	1.18	23	2.0	-	-
3/60	10/200	1.30	71	6.3	2	0.2
2/60	20/600	1.48	81	7.1	6	0.5
1/60	20/1200	1.78	87	7.7	4	0.4
CF	20/2000	2.10	5	0.4	-	-
HM	20/20,000	2.40	97	8.6	4	0.4
LP	-	2.70	85	7.5	1	0.1
Total			1133	100.0	1133	100.0

**Figure 1:**
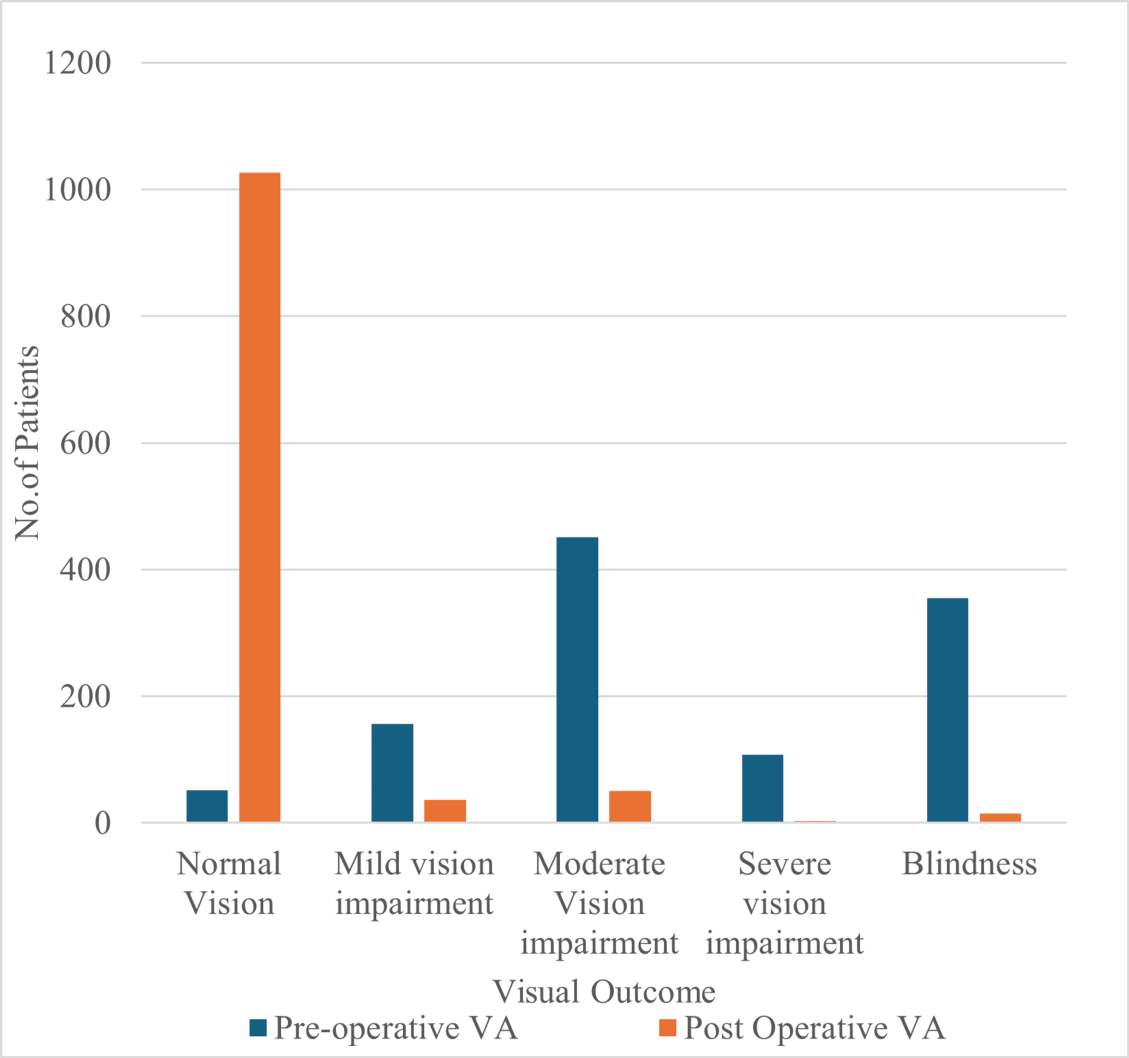
Distribution of Preoperative and Fourth-Week Postoperative Visual Acuity (VA) Based on the WHO Visual Impairment Criteria

Of the 1,133 patients, preoperatively, most had moderate vision impairment (n = 461, 40.7%), followed by severe visual impairment (n = 108, 9.5%) and blindness (n = 355, 31.3%), with only 52 (4.6%) patients having normal vision. At the fourth week postoperative follow-up, the majority achieved normal vision (n = 1,027, 90.6%), while a few remained with mild (n = 37, 3.3%), moderate (n = 51, 4.5%), severe (n = 3, 0.3%), or blindness (n = 15, 1.4%) visual impairment (Figure 1).

Among the 1,133 eyes operated, 1,027 (90.6%) patients achieved good outcomes, 88 (7.8%) had borderline outcomes, and 18 (1.6%) had poor outcomes (Table 3).

**Table 3:** Visual Outcomes of Cataract Surgery at Fourth-Week Follow-Up (n = 1,133)

Visual Outcomes	No. of Patients, n (%)
Good (≤0.3 logMAR)	1,027 (90.6)
Borderline (>0.3 to 1.0 logMAR)	88 (7.8)
Poor (>1.0 logMAR)	18 (1.6)

The effectiveness of cataract surgery, as measured by visual acuity outcomes at the fourth week after surgery, demonstrated a statistically significant increase in postoperative visual acuity. After surgery, mean visual acuity improved by 0.93 logMAR (SD = 0.71; p < 0.001), from 1.16 ± 0.69 logMAR preoperatively to 0.23 ± 0.26 logMAR postoperatively (Table 4).

**Table 4:** Effectiveness of Cataract Surgery on Visual Acuity Measured in logMAR Units (n = 1,133)

Visual Acuity	Preoperative	Fourth-Week Postoperative	Mean Difference (95% CI)	p-value
Mean (SD)	1.16 (0.69)	0.23 (0.26)	0.93 (0.71)	<0.001

Multivariable linear regression analysis revealed that age was significantly associated with visual acuity at the fourth week postoperative follow-up. Patients aged 60 years and above showed significantly less improvement in visual acuity than those below 60 years, with a p-value of <0.001. There was no discernible correlation between sex and post-operative visual acuity. Diabetes mellitus, however, showed a significant correlation with postoperative visual acuity (p = 0.014), with patients with diabetes reporting a lower gain in visual acuity than those without the disease. On the other hand, there was no significant correlation found between the changes in visual acuity and the presence of previous ocular comorbidities (Table 5). Only one patient (0.1%) experienced a corneal-related post-surgical complication, and no other postoperative complications were reported in this study.

**Table 5:** Distribution of Preoperative and Postoperative Visual Acuity by Age, Sex, Diabetes Status, and Previous Ocular Comorbidities (n = 1,133)

Category	Mean Change (in logMAR), Mean (SD)	Adjusted β (95% CI)	p-value
Age group			
Below 60 years	1.06 (0.72)	REF	<0.001
60 years and above	0.88 (0.69)	-0.12 (-0.89 to -0.27)	
Sex			
Male	0.90 (0.68)	REF	0.375
Female	0.95 (0.72)	0.03 (-0.05 to 0.12)	
Presence of diabetes			
Yes	0.85 (0.67)	-0.07 (-0.20 to -0.02)	0.014
No	0.97 (0.72)	REF	
Presence of previous ocular comorbidities			
Yes	0.73 (0.70)	-0.03 (-0.43 to 0.14)	0.316
No	0.94 (0.71)	REF	

## Discussion

Cataract surgery is the most frequently performed eye procedure worldwide and is the primary method for restoring vision in people affected by cataracts. It is crucial to monitor postoperative outcomes to improve the quality of cataract surgery over time, as visual outcomes influence an individual’s visual function and independence, thereby enhancing quality of life. To the best of our knowledge, no studies have been conducted in the Northern Sri Lankan region to evaluate visual acuity outcomes after cataract surgery. The present study evaluated postoperative visual outcomes in 1,133 patients or eyes who underwent cataract surgery at the Eye Unit of Teaching Hospital Jaffna.

The study found that cataract procedures using phacoemulsification with IOL implantation resulted in a significant improvement in post-operative visual acuity, with the primary outcome being BCVA in the operated eye at the fourth week after surgery. Our findings revealed good outcomes in 1,027 (90.6%), poor outcomes in 18 (1.6%), and moderate outcomes in 88 (7.8%), all in accordance with WHO guidelines for cataract surgical outcomes. These findings surpass the WHO's minimal recommendation, which states that at least 80% of operated eyes should achieve good postoperative vision with correction. The significant increase in visual acuity from 4.6% preoperatively to 90.6% postoperatively demonstrates both the technical success of the surgery and the adequacy of the refractive correction. Preoperatively, the majority of patients were classified as having moderate to severe visual impairment or blindness. After cataract surgery, there was a marked improvement in vision, with over 90% of patients achieving normal vision and a substantial decrease in the proportion of patients who remained blind. This further emphasises the effectiveness of phacoemulsification cataract surgery in a resource-constrained setting for functional vision restoration and for meeting the WHO-recommended standards for surgical outcomes in high-income countries.

Our findings are similar to those of a study conducted in Pakistan by Hashmi et al. (2013), who reported that 93.3% of operated eyes achieved good visual outcomes with phacoemulsification [17]. However, our results were better than those reported in the Sri Lankan National Survey by Murthy et al. in 2018, which reported a 75.1% good visual outcome rate [13]. The clinical effectiveness of cataract surgery in this population is further confirmed by the statistically significant improvement in mean visual acuity (mean difference 0.93 logMAR units, p < 0.001). Such outcomes are better than those reported in cataract-related studies conducted in adjacent countries such as Bangladesh, India, and Pakistan [18-20]. Further, Han et al.’s systematic review of 31 studies found that all five high-income countries' cross-sectional studies reported postoperative presenting visual acuity ≥0.32 in over 70% of participants, which aligns with the findings of this study [21].

The number of cataract surgeries was higher among patients aged 60 years and above. This finding is consistent with previous studies conducted in developing countries [22-24]. Nevertheless, even in this age group, postoperative visual outcomes remained good, underscoring the appropriateness of the standard of care across age groups. Cataract surgery rate was found to be greater in female patients than in male patients, which was also evidenced in several studies [25]. However, sex, religion, job status, ethnicity, side of eye operated, and previous ocular comorbidities did not have a significant effect on postoperative visual improvement, similar to existing studies that have reported no significant difference in visual outcome based on sex [26].

Patients with diabetes mellitus were found to have a significant association with less postoperative visual gain (adjusted β at 95% CI, -0.07 (-0.20 to -0.02); p < 0.001), which is comparable to a large multicentre study of 90,729 eyes, showing mean postoperative visual acuity was worse in people with diabetes (0.23 vs. 0.13 logMAR; p < 0.0001) [27]. However, diabetic patients still achieved a good visual improvement. This may reflect the impact of diabetic retinal changes, subclinical macular oedema, or delayed visual recovery, even in the absence of overt diabetic retinopathy [26].

In our study, patients with normal vision were also included, as those who needed surgery showed functional impairment. This highlights that access to cataract surgery should not be restricted by visual acuity, as the threshold for cataract surgery is often set at 6/9 or better, and provides evidence that visual function improves after cataract surgery among patients with preoperative visual acuity of 6/9 or better. Further, postoperative complications in this study were less than 0.1%, which is much lower than the rates reported in other studies, indicating the safety and effectiveness of the surgical techniques. The poor outcome of 1.6% reported in this study could be due to the presence of other ocular comorbidities and systemic comorbidities [28].

Overall, the study highlights that cataract surgery at the Eye Unit of Teaching Hospital Jaffna delivers excellent visual outcomes that meet and surpass international benchmarks. However, this study has several limitations. First, the retrospective design may add selection bias and restrict control over data completeness, as the research depended on information documented in current medical records. Second, a four-week follow-up period may not adequately capture long-term vision results or late postoperative problems, which can emerge several months following cataract surgery. This short follow-up was attributed in part to the study's context, in which a major proportion of the patient population is predominantly rural, making long-term follow-up assessments challenging. As a result, the study focused on the fourth week postoperative follow-up visual outcomes as supported by a previous study by Deshpande et al. (2021) [29]. Further, lack of uncorrected visual acuity and patients' reported outcome measures limit a more comprehensive assessment of functional and long-term quality outcomes. As the study was conducted in a resource-constrained setting, addressing these gaps could further elevate the quality of care.

## Conclusions

This retrospective analytical study, carried out in a resource-constrained, lower-middle-income setting, demonstrated that phacoemulsification with IOL implantation led to a significant visual improvement at the fourth week postoperatively, with 90.6% of patients achieving a BCVA of 6/12 or better, consistent with WHO visual outcome benchmarks reported in developed countries. Even though older age and the presence of diabetes mellitus were associated with postoperative visual outcomes, the proportion of poor outcomes (1.6%) and postoperative complications (>0.1%) was low compared with studies from other countries. Notably, despite the expected influence of age, our findings reveal that patients across all age groups achieved good postoperative visual outcomes following cataract surgery, highlighting the effectiveness and safety of phacoemulsification even in a resource-constrained setting.

However, given the retrospective design and relatively short follow‑up period of four weeks, these findings primarily reflect short‑term outcomes and cannot capture longer‑term visual results or late complications. Within these limitations, our findings highlight the feasibility of delivering high‑quality cataract surgical services in resource‑constrained settings and support continued investment in phacoemulsification to alleviate avoidable visual impairment and blindness, enhance quality of life, and reinforce equitable eye care delivery in similar contexts while highlighting the need for future prospective studies with longer follow‑up and patient‑reported outcomes to further validate these findings.
